# Pleural Effusion with Mediastinal and Hemidiaphragm Mass Effect: A Case Report

**DOI:** 10.7759/cureus.5372

**Published:** 2019-08-12

**Authors:** Rachel E Bridwell, Michael J Yoo, Joshua J Oliver

**Affiliations:** 1 Emergency Medicine, Brooke Army Medical Center, Fort Sam Houston, USA; 2 Emergency Medicine, San Antonio Uniformed Services Health Education Consortium, Fort Sam Houston, USA

**Keywords:** pulmonary effusion, leiomyosarcoma, metastases

## Abstract

Pulmonary metastases are a rare but aggressive and life-threatening complication of leiomyosarcoma. We discuss a case of a 48-year-old woman with stage 4b leiomyosarcoma who presented with dyspnea and hemodynamic instability secondary to a large lung metastasis with massive pleural effusion. This particular subset of patients is vulnerable to re-expansion pulmonary edema in a disease with poor survival rates.

## Introduction

Leiomyosarcomas are rare but aggressive soft tissue tumors associated with a poor prognosis and affecting approximately 0.36 per 100,000 women-years [1]. These tumors typically present with abnormal uterine bleeding and early metastases to the lung. There is one pulmonary leiomyosarcoma per 3000 pulmonary carcinomas reported [2]. However, minimal case studies document significant cardiopulmonary complications secondary to pulmonary metastases and subsequent cardiovascular compromise. We discuss a 48-year-old woman with a history of metastatic leiomyosarcoma who presented to the emergency department with acute dyspnea, hemoptysis, and hypotension and was found to have a large left-sided pleural effusion causing mediastinal, hemidiaphragmatic, renal, and splenic shift.

## Case presentation

A 48-year-old woman with a history of stage 4b leiomyosarcoma with lung metastases diagnosed in 2016 status post-hysterectomy and on active doxorubicin and dacarbazine presented to the emergency department (ED) with acute shortness of breath and scant hemoptysis. On a further review of systems, the patient endorsed dyspnea on exertion that improved with rest and upright position, orthopnea, paroxysmal nocturnal dyspnea, and subjective fevers. She denied chest pain, diaphoresis, or chills. Further history and chart review revealed known lung metastases to include: an 8.9 x 8.5 x 6.3 cm posterior left lower lobe complex mass, 1.3 cm left upper lobe solid nodule, and a 6 mm right upper lobe subpleural pulmonary nodule. These findings were identified on computed tomography (CT) of the chest performed three weeks prior to presentation. The patient had been in her baseline state of health prior to this ED visit and underwent her scheduled doxorubicin and dacarbazine infusion two days prior.

**Figure 1:**
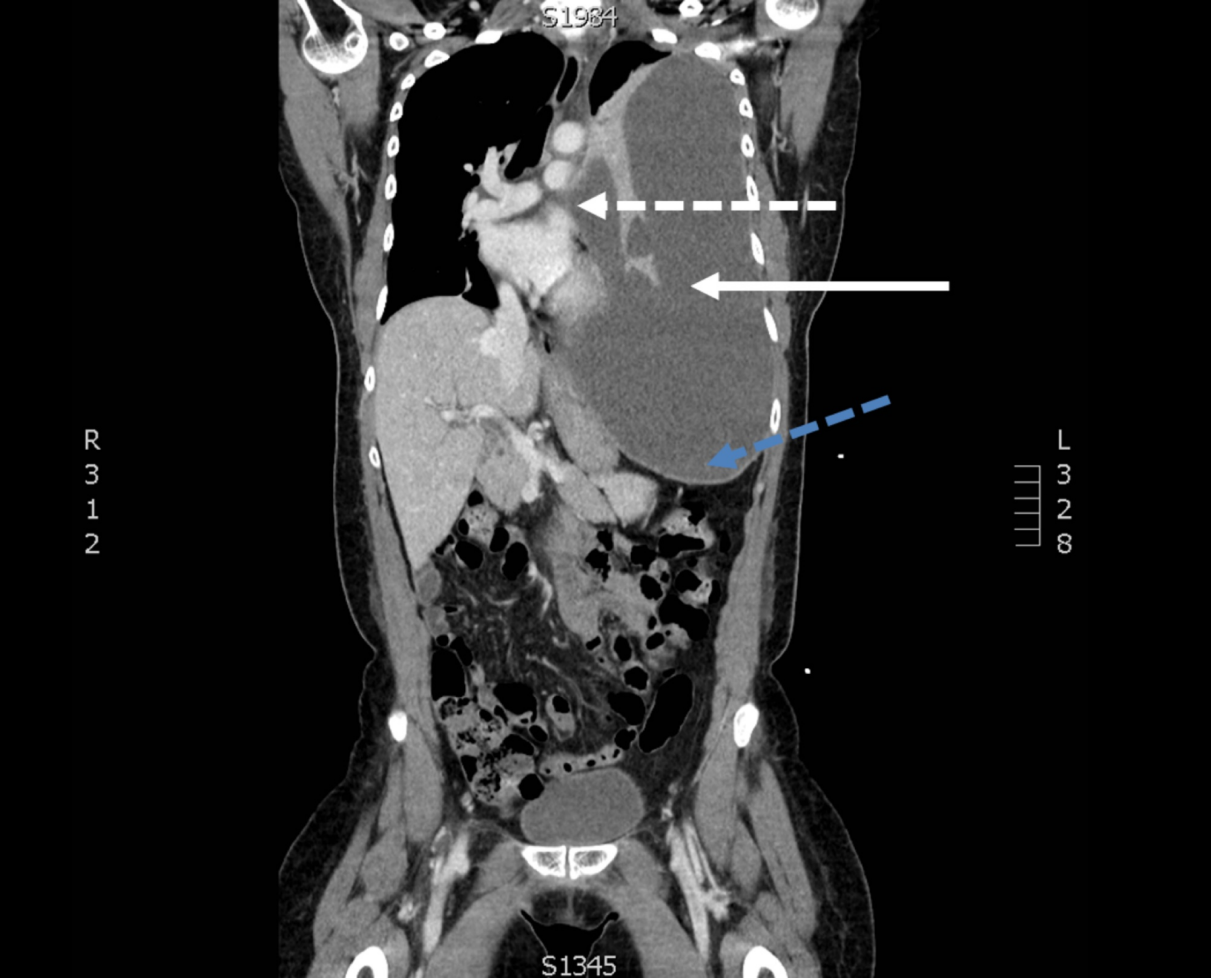
A coronal slice of a contrasted computed tomography of the chest, abdomen, and pelvis demonstrating a large left-sided pleural effusion (solid white line) with mass effect on the mediastinum (dashed white line) and left hemidiaphragm (dashed blue line)

**Figure 2:**
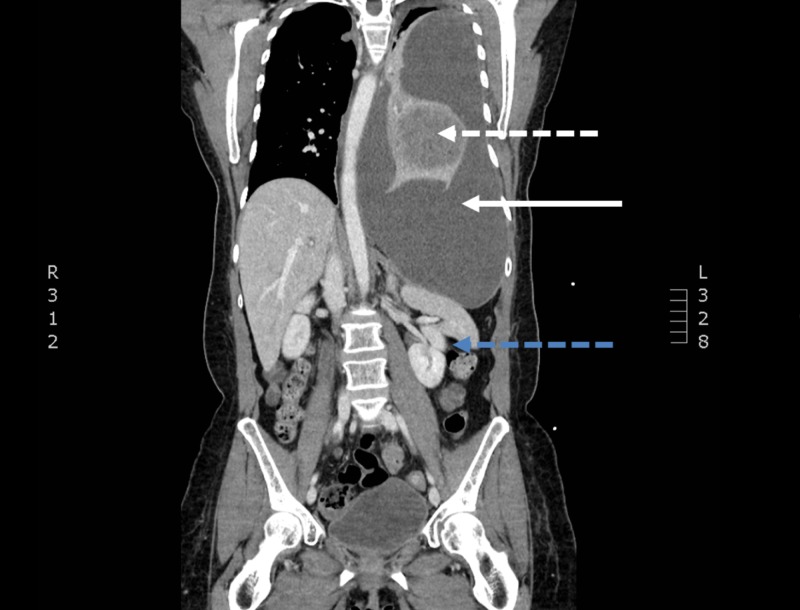
A coronal slice of contrasted computed tomography of the chest, abdomen, and pelvis demonstrating a complex mass (dashed white line) with associated large left-sided pleural effusion (solid white line) with mass effect on the left hemidiaphragm, left kidney, and spleen (dashed blue line)

On arrival, the patient’s vital signs included: heart rate of 133 beats per minute (bpm), blood pressure of 69/50 mmHg, temperature of 101.1⁰ Fahrenheit, and pulse oximetry of 95% on room air. The patient’s physical exam was remarkable for conjunctival pallor, decreased breath sounds, and dullness to percussion on the patient’s right posterior lung fields. Intravenous access was established, and the patient received one unit of fresh whole blood followed by 2 liters of lactated Ringer’s solution and 1 gram of cefepime. The patient’s blood pressure and heart rate improved to 110/60 mmHg and 110 bpm, respectively. A subsequent contrasted CT of the chest, abdomen, and pelvis demonstrated a large left pleural effusion causing a nearly complete collapse of the left upper and lower lung. Mass effect was also noted, to include a rightwards mediastinal shift and a downwards left hemidiaphragm shift displacing the spleen and left kidney inferiorly (Figures 1-2). Serum studies revealed 350 white blood cells per mm of blood, 35 neutrophils per mm of blood, and a platelet count of 17,000. The patient was administered six units of platelets and admitted to the medical intensive care unit (MICU) where 2 liters of sanguineous pleural fluid were drained with thoracentesis.

## Discussion

Leiomyosarcomas comprise 0.5% of lung neoplasms and can seed the lungs, remaining dormant for many years [3-4]. While the patient exhibited cardiorespiratory collapse secondary to mediastinal shift and left lung collapse, pulmonary metastases may also demonstrate left atrial extension, causing heart failure and pulmonary hypertension. These potential complications can create a clinically challenging scenario, necessitating the balance between low tidal volumes and inotropy [5]. While no large case series for pulmonary leiomyosarcomas exists, limited publications report a similar presentation of severe dyspnea and hemodynamic instability from massive pulmonary effusion [6-7].

In considering thoracentesis for diagnostic and symptomatic relief, no more than 2000 mL should be aspirated to avoid re-expansion pulmonary edema, a potentially lethal complication [8]. Although the exact pathogenesis of this phenomenon is unclear, rapid re-expansion, prolonged duration of lung collapse, decreased surfactant activity, and increased pulmonary vascular permeability secondary to micro-vessel injury appear to be contributing risk factors [9-11]. In the setting of malignant pleural effusion, especially in patients with metastatic leiomyosarcoma, judicious aspiration and possible manometry are critical.

If amenable to operative management, including patient preference and cancer grading and staging, surgical resection is the mainstay of treatment, with chemotherapy and radiation as adjuvant options [12]. However, if the tumor is unresectable, leiomyosarcomas have been shown to be sensitive to doxorubicin, ifosfamide, trabectedin, with a general response rate of 20%; in these cases of large tumor burden and progressive disease, the median survival rate is estimated to be 12 months [13-14].

## Conclusions

Pulmonary metastasis is a rare but immediately life-threatening complication of leiomyosarcoma. Pleural effusion and mass effect can cause both cardiorespiratory collapse while aggressive thoracentesis can further complicate resuscitation. Surgery with or without adjuvant therapy remains the mainstay of treatment in this aggressive cancer with poor survival rates.
